# One of These Things Is Not Like the Others: Disentangling the Concepts of Range of Motion Versus Flexibility, and Flexibility Training Versus Stretching

**DOI:** 10.1007/s40279-026-02425-4

**Published:** 2026-03-25

**Authors:** José Afonso, Anthony J. Blazevich, David George Behm, Markus Tilp, Konstantin Warneke

**Affiliations:** 1 Faculty of Sport, Centre of Research, Education, Innovation, and Intervention in Sport (CIFI2D), University of Porto, Porto, Portugal; 2 School of Medical and Health Sciences, Edith Cowan University, Joondalup, Australia; 3 School of Human Kinetics and Recreation, Memorial University of Newfoundland, St. John’s, NL Canada; 4 Department of Human Movement Science, Sports and Health, University of Graz, Graz, Austria; 5 Institute of Sustainability Education and Psychology, Department for Research Methods and Evaluation, Leuphana University Lüneburg, Lüneburg, Germany

## Abstract

In this Current Opinion, we revisit key terminological distinctions in sports sciences, emphasizing the implications of conflating related but distinct concepts, particularly range of motion (ROM) versus flexibility and flexibility training versus stretching. While often used interchangeably, these terms represent nuanced constructs with distinct physiological and practical implications. ROM encompasses both modifiable (e.g., soft tissue extensibility, neural control) and non-modifiable factors (e.g., bone structure), whereas flexibility is only one of several trainable components of ROM, largely referring to the extensibility of periarticular soft tissues. Misunderstanding these distinctions risks erroneous assessments and inappropriate exercise prescriptions. We also challenge the common practice of equating flexibility training with stretching. While stretching is effective to enhance flexibility, other methods, such as strength training at long muscle lengths and foam rolling, produce comparable chronic ROM gains. Furthermore, stretching has a diverse array of effects that extend beyond flexibility, including strength development and neural modulation. Mischaracterizing flexibility training as synonymous with stretching perpetuates myths and constrains innovation in training practices. By differentiating these terms, we advocate for clearer language in research and practice to avoid miscommunication and ensure effective training interventions. In summary, we propose that flexibility is only one component of ROM, and that flexibility training and stretching should not be equated. We hope this Current Opinion paper generates a healthy discussion within the sports science and medicine communities.

## Key Points

**Table:** 

ROM and flexibility are distinct constructs: ROM is broader and includes modifiable factors such as soft tissue extensibility and neural control, but also (mostly) non-modifiable elements such as bone structure and joint anatomy. Flexibility is a trainable component of ROM.
Flexibility training is not limited to stretching—flexibility can be improved through stretching, but also through methods such as strength training and foam rolling.
Stretching affects more than just flexibility (e.g., strength gains, neural modulation), thus its applications should not be limited to flexibility development.

## All Things Are Not the Same

Sports sciences should strive to clearly demarcate concepts that should not be confused or conflated. Not in the name of “terminological purity,” but to avoid conveying the wrong messages to a broader audience. Cases in point include the terms range of motion (ROM) versus flexibility and flexibility training versus stretching. By interchangeably using the terms ROM and flexibility, we are neglecting non-modifiable factors influencing ROM, potentially under- or over-doing flexibility training, or sometimes forcing the joints too far (e.g., in passive or assisted stretching). We may also risk neglecting the role played by other modifiable factors traditionally not associated with flexibility (e.g., balance and pain, as will be addressed in Sect. 2).

By equating flexibility training with stretching, we are conveying the (wrong) idea that primarily or only stretching is required to improve flexibility although alternatives exist. Simultaneously, we are potentially forgetting that stretching can have other applications (e.g., strength gains) that are not limited to improving flexibility (or not mediated by increased flexibility). But let’s start from the beginning. Our goal is to discuss ROM and flexibility as well as flexibility training and stretching, building the case that these pairs of concepts should not be equated or used interchangeably (i.e., ROM ≠ flexibility, flexibility training ≠ stretching) and that their differentiation has relevant implications for how practitioners interpret them and therefore how they affect daily practices. We will start from definitions provided by major world-renowned institutions, as these are likely to have greater impact than isolated scientific publications.

## ROM Versus Flexibility: They Are Not the Same

The National Strength and Conditioning Association (NSCA) defines ROM as “the amount of rotation available at a joint” (p. 276) [1]. Similarly, the 11th edition of the American College of Sports Medicine (ACSM) Guidelines for Exercise Testing and Prescription initially defines flexibility as “the ROM available at a joint” (p. 28^1^) [2], and then as “the ability to move a joint through its complete, *pain-free* ROM” (p. 104) [2]. In the same vein, the National Academy of Sports Medicine (NASM) defines flexibility as “the ability to move a joint through its complete ROM” (p. 162) [3]. The NSCA, however, establishes *mobility* as the ability to move through full ROM, and *flexibility* as the extensibility of the periarticular soft tissues, considering flexibility as only one of several factors limiting ROM (p. 275) [1]. Given the very heterogeneous use of the word “mobility,” which can be very broad (e.g., the timed up-and-go test [4]), we will not further discuss this concept here. Similar to the NSCA, peer-reviewed publications often use “flexibility” and “extensibility” interchangeably [5–7]. Finally, a very popular book on training methodology defines flexibility as “the ROM of a joint or set of joints” (p. 236) [8]. The World Health Organization was not cited because their latest guidelines for physical activity do not address these topics [9], and we also failed to find relevant material from the International Olympic Committee, which was surprising.

Across its guidelines, the ACSM interchangeably mentions measurement of flexibility and measurement of ROM, and at a certain point states that flexibility can be quantified in terms of ROM expressed in degrees (p. 105) [2]. The NSCA has a similar approach (despite the previously mentioned distinction between mobility and flexibility), and goes as far as equating ROM tests to measuring muscle length (p. 286) [1]. Bompa and Buzzichelli [8] (p. 326) also state that muscle length is what limits ROM (if only it were that simple…). However, the ACSM guidelines also state that “flexibility training is important to enhance ROM” (p. 262) [2], implying that flexibility can be trained and is therefore modifiable. Similarly, flexibility has been considered a trainable biomotor ability [8], meaning that motor learning and not only physiological or biomechanical adaptations can take place. This raises the question: Is ROM fully modifiable, or may it be limited by non-modifiable factors (e.g., bone and joint structure), and if so, should it be equated with flexibility? Or should flexibility be considered only a modifiable (and therefore trainable) part of ROM?

First, it should be acknowledged that there are *non-modifiable* components of ROM, namely bone and joint structure [1], which have relevant inter- and even intra-individual variations that affect ROM [10] (e.g., knee and elbow extension, or less evident features, such as an *os talus secundarius*, limiting subtalar ROM and causing restriction and pain in sports activities [11]). The case of elbow extension can be easily observed, as variation in bone shape and size limits this action differently from person to person [10]. A typical case affecting athletes is femoroacetabular impingement syndrome, which (regardless of being a natural anatomic variation or acquired due to repetitive movements) negatively affects hip flexion, adduction, and/or internal rotation and commonly requires arthroscopic intervention [12, 13].

Body-on-body contact (e.g., knee flexion, where the movement will stop upon contact of the posterior aspects of the leg and thigh) will also limit ROM, acting as a physical barrier [1] regardless of flexibility levels. Body size ratios may influence distance-based assessments (e.g., inhibition of sit-and-reach distance by a protuberant or distended waistline) but likely play a minor role in angle-based assessments (e.g., through goniometry). If ROM is influenced by both modifiable and non-modifiable factors, and flexibility is a modifiable factor [1–3, 8], then it is reasonable not to equate flexibility with ROM, and instead consider it only as one of its components.

However, flexibility as the extensibility of soft tissues [1, 5–7] (and considered trainable by most accounts [1–3, 8]) is not the only *modifiable* component limiting ROM. The ACSM considers flexibility a health-related physical fitness component, while it catalogs balance under skill-related physical fitness components (p. 28) [2]. However, a study asked 20 healthy young adults (14 women) to perform the toe-touch test (also known as the stand-and-reach) under standard conditions versus using a device restraining the participant against forward falls [14]. When restrained (i.e., when risk of falling was removed through use of a Velcro strap attached to a platform support), the participants showed superior ROM to the traditional toe-touch test (− 4.1 ± 4.9 cm versus − 15.2 ± 6.5 cm, respectively), and therefore the authors suggested that balance influenced flexibility [14]. Alternatively, it could be stated that fear of falling (and not balance per se) was responsible for the differences, or that using the Velcro strap may have induced postural changes and influenced ROM. Regardless, it suffices to establish that “flexibility” is not the single modifiable factor limiting ROM.

The NASM defines optimal control of movement through full ROM as dynamic ROM, and states that it depends on a mixture of flexibility and neural control of movement (p. 163) [3], and this includes muscle recruitment and balancing strength production across agonists, antagonists, synergists, and stabilizers (p. 163) [3]. Therefore, achieving full ROM relies not only on flexibility (i.e., soft tissue extensibility [1, 5–7]), but also on accurately managing force production, which are the typical tenets of strength training. Perhaps unsurprisingly, strength training (at least when performed to relatively long muscle lengths) has been shown to improve ROM with a magnitude comparable to that induced by stretching protocols [15–17], and suggests that ROM is limited by much more than soft tissue extensibility*.* In fact, active stretching through eccentric resistance training seems quite effective for generating ROM gains [18], with the “active” component again hinting at a large role played by neural modulation and not merely tissue extensibility.

Beyond these factors, pain can also negatively affect ROM acutely [19], which may affect athletes’ ROM after injury or surgery, for example. There is substantial variability between people in their pain tolerance and even within individuals over time (e.g., before or after exercise). Thus, is ROM primarily dependent upon how much or little discomfort someone can tolerate? It is not our intention to be exhaustive in the context of this Current Opinion article, but merely to illustrate that ROM is a wider concept than “flexibility” (in agreement with the NSCA’s proposal [1]), which is just one piece of the ROM puzzle that is more related to soft tissue (e.g., muscle, tendon, nerves) properties. Several other modifiable and non-modifiable factors influence ROM.

This distinction between flexibility and ROM has implications for the reporting of scientific findings. ROM is a measurable property—there is a motion, and that motion can be performed throughout a given range. ROM is thus an objective parameter that can be assessed externally and can be limited by factors unrelated to flexibility (e.g., bone on bone contact, typical in elbow extension). On occasion, ROM may be inferior to the available flexibility: beyond elbow extension (limited by the configuration of bony articular surfaces), a deep squat is a movement that will inevitably stop when the thigh contacts the lower leg, even if the person would theoretically have the flexibility to perform a deeper movement. We propose that studies should report ROM, as this is the objective measure that is most commonly assessed (e.g., using goniometers or inclinometers [20]) and reported (e.g., in degrees or radians). Flexibility would require assessments of soft tissue extensibility, such as ultrasound testing of fascicle length elongation [21, 22]. However, these assessments require laboratorial settings and may be impossible to perform in vivo. All in all, most assessments will in practice reflect ROM, not necessarily flexibility, thus this term should probably be used by default.

## Flexibility Training Versus Stretching: Two Very Different Things

The ACSM equates “flexibility training” with stretching exercises [2]. Indeed, box 5.5 titled “Flexibility Exercise Definitions” only considers stretching exercises (p. 148) [2], and this limitation to stretching exercises is reinforced later in the text (page 149). Likewise, the NASM only considers stretching when referring to flexibility training, although they categorize self-myofascial release (a misnomer in and of itself; see [23]) as a form of stretching (pp. 173, 174) [3]. Finally, the NSCA also postulates that ROM gains are achieved via stretching (p. 291) [1]. This raises the question: Are flexibility training and stretching the same thing? Or can flexibility be improved by other training methods?

First, it is important to realize that flexibility is a trainable fitness component or ability [1–3, 8], which can be developed through different means. A widely popular book on training methodology stated that strength training through a full ROM would result in ROM gains [8]. Because these gains would likely not be at the expense of non-modifiable factors (that would probably result in an injury), and the statement is broad (i.e., strength training in general, meaning that balance does not need to play a large role in the selected exercises), it may be speculated that strength training (but not calisthenic resistance exercises [15]) may improve ROM through improved flexibility (i.e., soft tissue extensibility [1, 5–7]), as well as through better neural regulation of force production [3]. Recent meta-analyses confirm that strength training chronically improves ROM with comparable magnitude to stretching [15, 16]. Potentially, stretching and strength have similar underlying mechanisms (e.g., mechanical tension produced through elongation of the muscles). Therefore, strength training (including, but not limited to, eccentric training) could potentially be considered a form of flexibility training. In fact, the concept of “loaded stretching” clouds the difference between stretching and strength training [24] (also a discussion for the future). In addition, foam rolling has shown similar chronic ROM gains to stretching [25].

Acute ROM gains can be obtained through a plethora of methods (including the aforementioned stretching methods and strength training). For example, foam rolling [26–28] and a general warm-up [29] have shown similar acute ROM gains to stretching. In the absence of evidence regarding chronic ROM gains, different methods should probably not be considered “flexibility training,” as it is not clear they will improve soft tissue extensibility over time. However, there is evidence for chronic ROM gains when using foam rolling [25]*.* Overall, improving flexibility training does not necessarily require stretching, as this is only one possibility to achieve that goal [30]. As a side-note, given the previously mentioned definition of flexibility involving “*pain-free* ROM” (p. 104) [2], should this exclude methods such as passive/assisted stretching and proprioceptive neuromuscular facilitation (p. 148) [2], whereby an external partner or machine aims to go beyond the normal passive ROM?

**Fig. 1:**
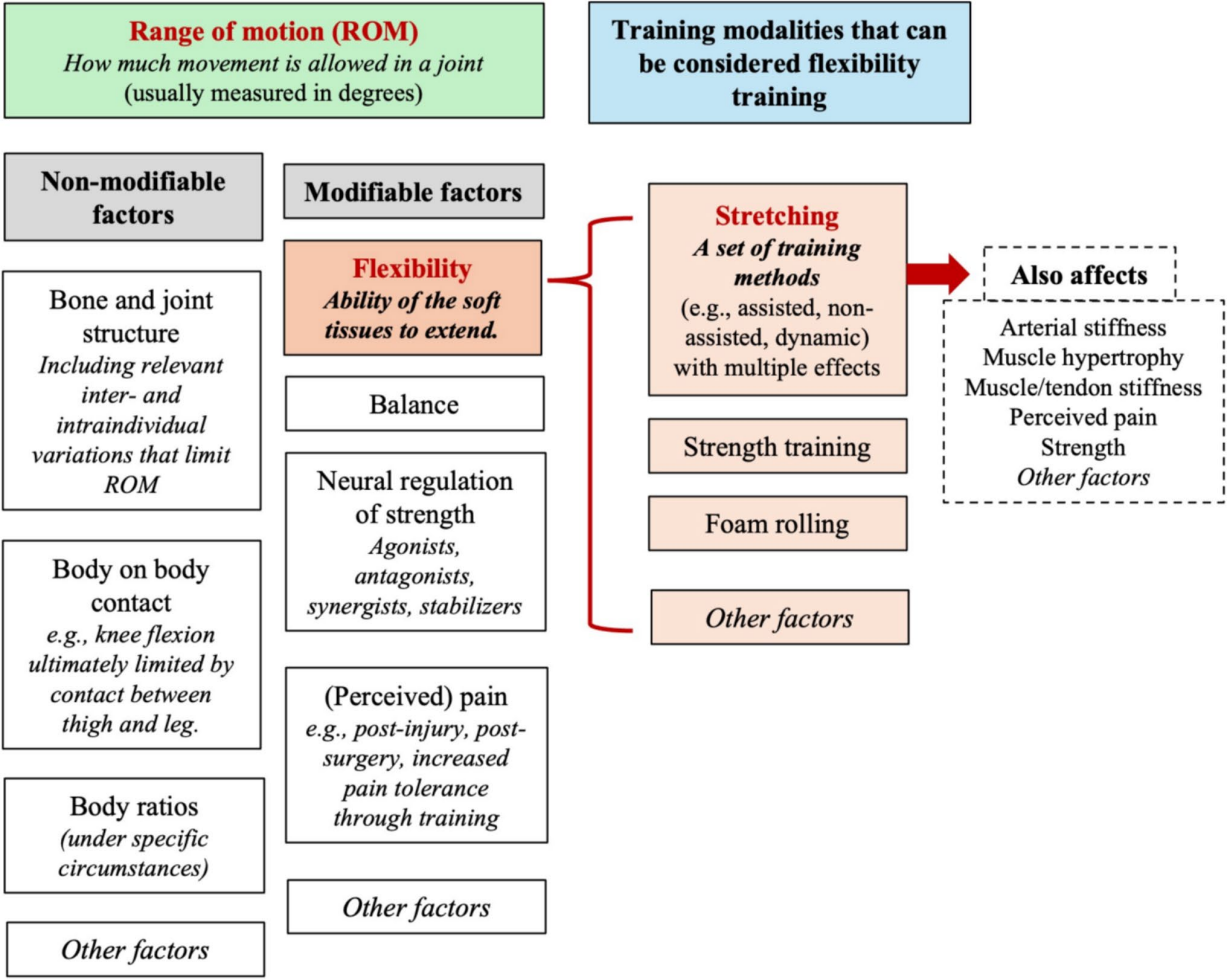
Scheme illustrating the relationships between range of motion (ROM), flexibility, flexibility training, and stretching

Second, stretching is a training method (albeit with different “flavors,” e.g., assisted (passive) static stretching, ballistic stretching), and therefore its effects are not limited to improving flexibility (in the sense of enhanced soft tissue extensibility [1, 5–7]). Indeed, stretching has demonstrated effects in promoting muscle hypertrophy and strength [31, 32]; reducing muscle [33, 34], nerve [35], and arterial stiffness [36, 37]; and reducing perceived pain [38], just to mention a few. Therefore, there exists no one-to-one correspondence between flexibility and stretching. Figure 1 synthesizes the key concepts presented throughout this manuscript.

## Conclusions and Ways Forward

Words carry meaning, and therefore discourse matters. Equating ROM (which has modifiable and non-modifiable components) with flexibility (a modifiable component) may lead to erroneous assessments of “flexibility deficits” and generate exercise prescriptions that may potentially violate the person’s natural ROM limits (especially if going over the limits, as may occur when using passive/assisted stretching). This extends to inter-limb asymmetries in ROM, which may result from asymmetrical bone or joint structures (e.g., due to natural intra-individual variations in bone size or angle [10] or previous injury [30]) and be unrelated to flexibility deficits. Such erroneous discourses also raise concerns of potential nocebo effects [39, 40]. Regardless of how flexibility is defined, considering it trainable means it must not be equated with ROM, as ROM depends on several modifiable and non-modifiable parameters. Therefore, research assessing ROM (e.g., degrees of shoulder flexion) should simply use the terminology ROM instead of flexibility, both in the title and throughout the manuscript.

In the same vein, the expression “flexibility training” may include stretching but also strength training [15, 16], foam rolling [25], and other training methods [17]. Therefore, authors should either refer only to the specific training method used (e.g., stretching) or refer to “flexibility training through… (add specific method here).” Otherwise, the myth may be reinforced that flexibility training is stretching, which is not supported by the literature. Relatedly, stretching may be useful for much more than merely improving flexibility.

Finally, practitioners should probably focus on the mechanisms underlying adaptations to different training methods, instead of associating each method with a narrow set of possible adaptations. For example, because stretching and strength training can both induce mechanical overload, that may explain previously mentioned findings, such as stretching also inducing strength grains and strength training inducing ROM gains. Moreover, terms such as “loaded stretching” blur the lines between stretching and strength training. In summary, this is a field where much work is still required and that is a great thing. We do not pretend to have arrived at the “ultimate definition” of these concepts, but we do hope this Current Opinion may contribute to a broader discussion within the sports science and medicine communities.

## Footnotes

1 All pages cited for the ACSM guidelines refer to the *epub* version, in which each page number corresponds to two pages or columns.
